# The First Clinical Case of Gorham-Stout Syndrome of Humerus in an 18-year-old Female Reported in Pakistan

**DOI:** 10.7759/cureus.4832

**Published:** 2019-06-04

**Authors:** Maratib Ali, Maryam Khan, Syeda Rida Abdi, Laila Tul Qadar, Mohammad Tahir Lakho

**Affiliations:** 1 Orthopaedics, Dr. Ruth KM Pfau Hospital, Karachi, PAK; 2 Miscellaneous, Dow University of Health Sciences, Karachi, PAK; 3 Internal Medicine, Dow Medical College and Civil Hospital Karachi, Dow University of Health Sciences, Karachi, PAK; 4 Internal Medicine, Dow University of Health Sciences, Karachi, PAK

**Keywords:** gorham-stout syndrome, vanishing bone disease, osteolysis, angiomatosis, lymphangiomatosis, humerus, gorham stout syndrome

## Abstract

Gorham-Stout syndrome (GSS) is a rare disorder of complete bone resorption, characterized by lymphangiomatosis and angiomatosis of bone, with only around 200 cases reported from around the world till date. The diagnosis is made on clinical, radiological, and histopathological findings and exclusion of other common conditions, and treatment is based on the physician’s judgment and tailored to the needs of the individual. With the etiology unknown, diagnosis mostly of exclusion, an unpredictable prognosis, and no standardized treatment formed, the disease poses a challenge to physicians in appropriately diagnosing and managing the patient. Herein, we present a case of an otherwise healthy 18-year-old female diagnosed with GSS of humerus following a fracture to her left arm. To our knowledge, this is the first case report of the disorder from Pakistan.

## Introduction

Gorham-Stout syndrome (GSS) was first reported by Jackson as 'a boneless arm' in 1838 and later detailed description was given by Gorham and Stout [1]. GSS is a rare disorder of unknown etiology, characterized by lymphangiomatosis and angiomatosis of bone leading to complete osteolysis, with only around 200 cases reported till date [2]. Clinical presentation is variable and the diagnosis, usually that of exclusion, is a challenge for physicians since the disease resembles other common conditions such as generalized lymphatic anomaly, fibrous dysplasia and osteomyelitis [3]. Misdiagnosis, hence, is not uncommon and adds to the disease burden. There is no standardized treatment available and the treating physician must turn to empirical data based on the limited literature present to provide the best possible management suited to the affected patient [4]. 

## Case presentation

**Figure 1:**
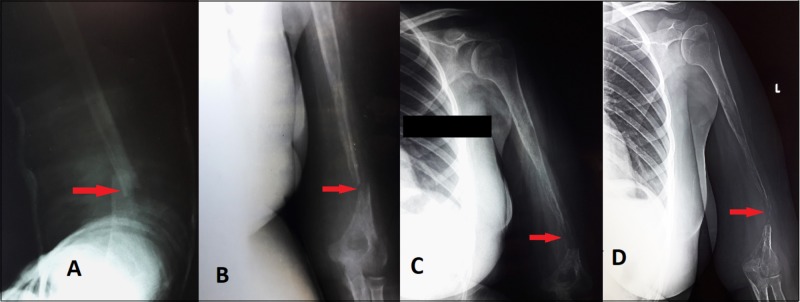
AP view plain radiographs of the left humerus taken on different occasions 1A) Plain X-ray AP view taken at a private clinic at the time of injury showing a simple transverse fracture at distal 1/3rd of the shaft of left humerus with displacement and angulation. 1B) Plain X-ray AP view after six weeks of injury showing the persistence of fracture with no callous formation indicating delayed union. Resorption of bone at the site of fracture is giving the typical ‘rat tail’ appearance of the shaft, osteopenia of the shaft is visible. 1C) Repeated plain X-ray AP view after three months of injury showing progressive resorption of bone. 1D) Plain Xray repeated on admission after five months of injury. AP view showing complete resorption of bone at the site of fracture with progressive thinning and disappearance of the shaft; generalized osteopenia of the bone is also visible. AP, anteroposterior

An otherwise healthy 18-year-old female was referred to the orthopedic unit of Dr. Ruth KM Pfau, Civil Hospital Karachi (CHK) for a non-healing fracture of the distal shaft of left humerus, sustained five months prior after falling on an outstretched arm. She was taken to the nearest hospital then, for left arm pain, where anteroposterior (AP) and lateral plain X-rays of left humerus showed a minimally displaced transverse fracture at the distal third shaft of the humerus (Figure 1A). The patient was managed conservatively with a Plaster of Paris (POP) back slab that was replaced by a POP full cast. However, X-ray performed after a period of six weeks showed no callus formation indicating delayed union along with the disappearance of the bone. (Figure 1B). Serial X-rays taken thereafter showed the persistence of fracture and bone resorption at the site along with generalized osteopenia in the shaft (Figure 1C). Hence, she was diagnosed with osteomyelitis and treated accordingly. Following no significant improvement of her symptoms and increased bone resorption at the fracture site as seen on X-rays, she was referred to our setup and was admitted for a detailed workup. A thorough evaluation was undertaken at our institution. A careful examination of the patient's left upper limb revealed global decreased muscle strength and limited motion of the left forearm. All active movements in the affected limb were mildly painful and limited at the shoulder and elbow joint, most notably flexion and extension. Wrist movements including gripping were normal. No neurologic deficits were found. A gap of nearly 3 cm was palpable in the distal part of the arm with palpable non-tender bony ends. Soft tissue swelling was absent and no lump was palpable along the length of the humerus. The skin overlying the fracture was normal. X-ray of the left arm was repeated, which showed marked disappearance of bone at fracture site resulting in a gap of 2-3 cm with tapering of bone ends and generalized osteopenia (Figure 1D).

There were no enlarged lymph nodes or any other abnormalities on the rest of her body. A complete blood workup ruled out hyperparathyroidism, renal osteodystrophy, osteomyelitis, vasculitis, etc. as secondary causes of bone resorption. Bone scintigraphy performed next with technetium 99m-methyl diphosphonate (99mTc MDP), showed mildly increased tracer uptake at distal 2/3rd of the shaft, ruling out our suspicion of malignancy. MRI scans revealed cortical and medullary erosion at left humeral shaft and complete resorption of the distal shaft with tapering of bone, indicating GSS. Consecutively, an incisional bone biopsy was sent for histopathologic evaluation, which demonstrated the increased proliferation of thin-walled vessels with no evidence of inflammatory or neoplastic cells, confirming GSS of the left humerus. The non-healing nature of the fracture, laboratory and radiological findings and histopathology exam fulfilled the criteria for Gorham disease suggested by Heffez et al. (Table 1).

**Table 1:** Criteria for diagnosing GSS proposed by Heffez et al. GSS, Gorham-Stout syndrome

	CRITERIA FOR DIAGNOSIS	FINDINGS PRESENT
1.	Positive bone biopsy	Yes
2.	Minimal or no osteoblastic response and no dystrophic calcification	Yes
3.	Non-expansive non-ulcerative lesion	Yes
4.	Absence of visceral involvement	Yes
5.	Osteolytic radiographic pattern	Yes
6.	Local progressive osseous resorption	Yes
7.	Absence of hereditary, metabolic, neoplastic, immunologic or infectious etiology	Yes
8.	Absence of cellular atypia	Yes

Consistent with the present literature on suitable treatment of upper extremity GSS, we planned to undertake a reconstructive surgery of left humerus using a fibular graft.

## Discussion

To date, only about 200 cases of GSS have been reported worldwide [2]. Herein, we present the first case of GSS encountered at our institution, and to our knowledge, this is also the first reported case of this disorder from Pakistan. GSS is classified as type-IV of the five types of primary idiopathic osteolysis described by Hardegger et al. and is characterized by proliferation of intra-osseous thin-walled vascular structures suggestive of lymphangiomatosis and/or hemangiomatosis, resulting in destruction and resorption of the osseous matrix [5-6]. No predilection for gender, race, and geography seems to exist. Generally, the patients present in the second to third decades of life but reports of age from one month to 75 years exist in the literature [7]. Pathogenesis of the disease is unclear; however, recent studies showing elevated levels of IL-6 point toward osteoclast as being one of the culprit causing osteolysis [8]. Furthermore, detection of immunohistochemical markers of lymphatic channels, notably LYVE-1 and podoplanin, suggests that lymphatic malformation is the primary pathology. However, the etiology is still speculative with no hereditary, neoplastic, or infectious link and about 50% of all patients present with an episode of trauma before diagnosis [2-3]. Our patient also presented with a history of fall five months prior to her diagnosis and gave no previous or family history of similar occurrences.

GSS is arduous to diagnose due to the involvement of any part of a skeleton, most commonly thorax, femur, mandible, and pelvis. Our careful literature search yielded five cases involving humerus along with 7.4% cases of osteolysis of shoulder girdle starting in the humerus. The symptoms depend on the affected bone and range from mild pain to spontaneous fracture. It is mostly a diagnosis of exclusion and requires a high-grade clinical suspicion, since there is no specific test or procedure to definitively diagnose GSS and requires integration of detailed clinical evaluation, radiologic and histopathologic testing, and work-up to rule out other possible causes of osteolysis such as hyperparathyroidism, osteomyelitis, generalized lymphatic anomaly, fibrous dysplasia, etc. [9]. In 1983, Heffez et al. formulated the eight-point diagnostic criteria, which is widely used today to assist in diagnosis [10]. Our presented case fulfilled all the eight criteria and diagnosis was based on thorough blood work to exclude other common conditions, evidence of progressive radiologic osteopenia over the course of five months, MRI scans indicating complete distal humeral bone resorption, and confirmatory histopathologic changes on biopsy.

Indeed, this case was a diagnostic dilemma due to its initial presentation as fracture non-union and indication towards osteomyelitis. This is a hardly diagnosed disease and the misdiagnosis adds to the disease burden, already significant due to the massive restriction the disease causes on the quality of life of patients. The immobility of the left arm of our patient for the last seven months has not only caused muscular atrophy and marked functional disability but multiple treatments at different hospitals as a result of misdiagnosis have resulted in serious financial burden on both the caregivers and the health care. Regrettably, standardized medical management of fractures had no effect and patient has gradually lost a major portion of the shaft of the humerus besides development of osteopenia owing to the progressive nature of the disease, suggesting a long term reduction in quality of life, which will financially inflict both the individual and the health care. The treatment, as suggested by existing literature based on case reports and small case series, is mostly empiric including medical, surgical and radiation or a combination of any of these. However, due to the absence of randomized and control studies, no standard guideline exists for treatment which is therefore tailored according to the patient’s needs. So, for an early resumption of the mobility of her left upper limb, we have planned a humeral reconstruction with a fibular graft and a regime of bisphosphonates thereafter, to ensure graft survival. 

## Conclusions

GSS is a rare disorder of spontaneous osteolysis of a bone of unknown etiology. With a variable presentation and highly unpredictable prognosis, it poses a diagnostic and therapeutic challenge to the physicians. Hence, until a definite molecular basis of this disease can be identified for a conclusive diagnosis and treatment, it is imperative to exchange experiences of effective diagnostics and treatment of this infrequent entity to improve the management and care of the affected patients.
